# Lysophosphatidic acids and their substrate lysophospholipids in cerebrospinal fluid as objective biomarkers for evaluating the severity of lumbar spinal stenosis

**DOI:** 10.1038/s41598-019-45742-7

**Published:** 2019-06-24

**Authors:** Kentaro Hayakawa, Makoto Kurano, Junichi Ohya, Takeshi Oichi, Kuniyuki Kano, Masako Nishikawa, Baasanjav Uranbileg, Ken Kuwajima, Masahiko Sumitani, Sakae Tanaka, Junken Aoki, Yutaka Yatomi, Hirotaka Chikuda

**Affiliations:** 1 Department of Orthopaedic Surgery, The University of Tokyo, Faculty of Medicine, Tokyo, Japan; 2 Department of Clinical Laboratory Medicine, The University of Tokyo Hospital, Tokyo, Japan; 3 Graduate School of Pharmaceutical Sciences, Tohoku University, Molecular and Cellular Biochemistry, Sendai, Japan; 4 Department of Anesthesiology and Pain Relief Center, The University of Tokyo Hospital, Tokyo, Japan; 5 Department of Pain and Palliative Medicine, The University of Tokyo Hospital, Tokyo, Japan; 6 Department of Orthopaedic Surgery, Gunma University, Graduate School of Medicine, Maebashi, Japan

**Keywords:** Diagnostic markers, Neuropathic pain

## Abstract

Lysophospholipids (LPLs) are known to have potentially important roles in the initiation and maintenance of neuropathic pain in animal models. This study investigated the association between the clinical severity of lumbar spinal stenosis (LSS) and the cerebrospinal fluid (CSF) levels of LPLs, using human samples. We prospectively identified twenty-eight patients with LSS and fifteen controls with idiopathic scoliosis or bladder cancer without neurological symptoms. We quantified LPLs from CSF using liquid chromatography-tandem mass spectrometry. We assessed clinical outcome measures of LSS (Neuropathic Pain Symptom Inventory (NPSI) and Zurich Claudication Questionnaire (ZCQ)) and categorized patients into two groups according to their severity. Five species of lysophosphatidic acid (LPA), nine species of lysophosphatidylcholine (LPC), and one species of lysophosphatidylinositol (LPI) were detected. The CSF levels of all species of LPLs were significantly higher in LSS patients than controls. Patients in the severe NPSI group had significantly higher LPL levels (three species of LPA and nine species of LPC) than the mild group. Patients in the severe ZCQ group also had significantly higher LPL levels (four species of LPA and nine species of LPC). This investigation demonstrates a positive correlation between the CSF levels of LPLs and the clinical severity of LSS. LPLs are potential biomarkers for evaluating the severity of LSS.

## Introduction

Lumbar spinal stenosis (LSS) is a common clinical condition presenting with neuropathic pain in elderly patients. Patients with LSS usually complain of low back pain and radiating lower leg pain, with or without foot numbness and dysesthesia. Some patients demonstrate motor weakness of the lower extremity and depressed tendon reflex. The anatomical severity of LSS is directly linked to symptom severity^1^, and surgical treatment of LSS generally produces favorable outcomes. However, 20% to 30% of patients who undergo surgery are dissatisfied with the results because of residual symptoms^2–5^. Despite the importance of surgical treatment, its indication typically relies on individual patient’s subjective symptoms, and imaging findings do not always accurately reflect the severity of their signs and symptoms^6–9^. Given the lack of reliable correlation between the severity of subjective and objective findings, the appropriate indications and optimal timing for surgical treatment of LSS are unclear.

Lysophospholipids (LPLs), such as lysophosphatidic acid (LPA), activate diverse groups of G-protein-coupled receptors and regulate decisive cellular functions. Among LPLs, LPA and lysophosphatidylcholine (LPC) have been well studied; each of these compounds share a simple chemical structure consisting of a 3-glycerol backbone with an attached single acyl chain of varied length and saturation, which determines the molecular species of LPA and LPC. LPA, structurally composed of a fatty acid linked to sn-glycerol-3 phosphate, is mainly converted from LPC via lysoPLD activity of autotaxin (ATX). It is suggested that LPA and LPC have key roles in the initiation and maintenance of neuropathic pain in animal models; blockade of their signaling is a potential therapeutic target in the prevention and treatment of neuropathic pain^10–13^, however the involvement of these lipid mediators in the pathogenesis of neuropathic pain has not been elucidated in human subjects. In a previous study, the plasma levels of the molecular species of LPLs, including unfamiliar LPLs such as lysophosphatidylserine and lysophosphatidylinositol (LPI), in human samples were accurately determined using liquid chromatography-tandem mass spectrometry (LC-MS/MS)^14^. However, the cerebrospinal fluid (CSF) levels of LPLs in humans have not been quantified, and the association between CSF levels of LPLs and the severity of neuropathic disorders has not been elucidated in clinical studies.

We used LC-MS/MS to measure LPLs species in samples obtained from the CSF of patients with LSS. Then, we analyzed the correlation between CSF levels of LPLs and the clinical severity of LSS.

## Results

### Patient characteristics

Twenty-eight patients with LSS were included (15 men and 13 women; mean age, 70.9 years; range, 49–81 years). All patients including controls completed the NPSI and ZCQ. In all control patients (n = 15), the NPSI score was 0 (no painful symptoms). For patients with LSS, those with an NPSI score less than or equal to the median value of 21 (n = 14) were categorized as the mild group and those with a score greater than or equal to 22 (n = 14) as the severe group. Concerning the NPSI subscore of paresthesia/dysesthesia, patients with LSS with a score less than the median value of 5 (n = 14) were categorized as the mild group and those with a score greater than or equal to 5 (n = 14) as the severe group. Concerning the ZCQ, patients with LSS with a score less than or equal to the median value of 20 (n = 14) were categorized as the mild group and those with a score greater than or equal to 21 (n = 14) as the severe group (Supplementary Fig. S1).

### LPL levels in CSF were higher in patients with LSS

Among all LPLs, five species of LPA (16:0, 18:0, 18:1, 18:2, and 20:4), and eight species of LPC (14:0, 16:0, 16:1, 18:0, 18:1, 18:2, 20:4 and 22:6) were detected in the CSF of all subjects. 22:6 LPA, 18:0 LPI, and 20:5 LPC were detected in the CSF of all patients with LSS and in 3 controls. ATX and LysoPLD activity were measured in the CSF of all patients, including controls, except for one patient with LSS due to insufficient sample volume. The CSF levels of all species of LPA, LPC, and LPI were significantly higher in patients with LSS versus controls (Table 1).

**Table 1:** Comparison of LPL levels in CSF between LSS patients and controls.

		LSS (n = 28)	Controls (n = 15)	*p-value*
LPA	Total	0.10 [0.067; 0.18]	0.034 [0.025; 0.049]	*0.001
	16:0	0.059 [0.043; 0.105]	0.021 [0.013; 0.031]	*0.01
	18:0	0.0056 [0.0033; 0.0081]	0.0018 [0.0007; 0.0024]	*0.032
	18:1	0.025 [0.012; 0.049]	0.0071 [0.0042; 0.0084]	*0.0007
	18:2	0.0045 [0.00069; 0.028]	0.00095 [0.00028; 0.0015]	*0.017
	20:4	0.0027 [0.00062; 0.0077]	0.00038 [0.00025; 0.0017]	*0.018
	22:6	0.00042 [0.00020; 0.0016]	0 [0; 0]	*0.026
LPC	Total	0.0054 [0.0038; 0.010]	0.0013 [0.0012; 0.0029]	*0.008
	14:0	0.00011 [0.000093; 0.00012]	0.000071 [0.000066; 0.000096]	*0.0003
	16:0	0.0017 [0.0012; 0.0037]	0.00043 [0.00033; 0.00091]	*0.0027
	16:1	0.000044 [0.000033; 0.000069]	1.4e-6 [3.8e-7; 0.000016]	*<0.0001
	18:0	0.0013 [0.00092; 0.0020]	0.00067 [0.00062; 0.00071]	*0.0044
	18:1	0.0014 [0.0010; 0.0021]	0.00013 [0.000089; 0.00068]	*<0.0001
	18:2	0.00041 [0.00023; 0.0010]	0.000029 [0.000017; 0.00012]	*0.0009
	20:4	0.00023 [0.00016; 0.00044]	0.000014 [6.5e-6; 0.00011]	*<0.0001
	20:5	0.000021 [9.5e-6; 0.000053]	0 [0; 4.0e-7]	*0.0088
	22:6	0.00015 [0.000085; 0.00029]	5.4e-6 [2.9e-6; 0.000053]	*0.0002
LPI	18:0	0.0018 [0.00061; 0.0043]	0 [0; 0.00021]	*0.02

Data are expressed as median value [25 percentile, 75 percentile], *p < 0.05.

LSS, lumbar spinal stenosis; LPA, lysophosphatidic acid; LPC, lysophosphatidylcholine; LPI, lysophosphatidylinositol; LPL, Lysophospholipid; CSF, cerebrospinal fluid.

### Some LPL species were positively associated with clinical parameters reflecting LSS severity

Patients in the severe NPSI group had significantly higher LPL levels (total LPC, three species of LPA, and nine species of LPC) versus the mild group (Table 2). Concerning the subscore of paresthesia/dysesthesia in NPSI, total LPC, one species of LPA, and eight species of LPC were significantly higher in the severe group versus the mild group (Table 3). Patients in the severe ZCQ group had significantly higher levels of LPLs (total LPA, total LPC, four species of LPA, and nine species of LPC) versus the mild group (Table 4). Regarding the association with morphological severity, total LPA, total LPC, LPI, four species of LPA, and nine species of LPC were significantly higher in the severe group versus the mild group (Table 5). Other LPL species also showed a trend towards higher levels in the severe group versus the mild group, both in terms of clinical and morphological severity, although these differences were not statistically significant.

**Table 2:** Comparison of LPL levels in CSF between severe and mild group with NPSI (total score) in the LSS patients.

		NPSI (Total score)		
		Severe (n = 14)	Mild (n = 14)	*p-value*
LPA	Total	0.130 [0.088; 0.29]	0.080 [0.057; 0.16]	0.052
	16:0	0.064 [0.047; 0.16]	0.051 [0.038; 0.098]	0.19
	18:0	0.0058 [0.0033; 0.0091]	0.0048 [0.0025; 0.0078]	0.35
	18:1	0.028 [0.018; 0.066]	0.020 [0.010; 0.037]	0.072
	18:2	0.027 [0.0029; 0.036]	0.0022 [0.00051; 0.0050]	*0.0044
	20:4	0.0050 [0.00071; 0.015]	0.0013 [0.00054; 0.0030]	*0.024
	22:6	0.00096 [0.00018; 0.0049]	0.00042 [0.00024; 0.00078]	*0.033
LPC	Total	0.0064 [0.0049; 0.0189]	0.0040 [0.0036; 0.0059]	*0.011
	14:0	0.00011 [0.00010; 0.00016]	0.00010 [0.000092; 0.00011]	*0.046
	16:0	0.0023 [0.0015; 0.0076]	0.0013 [0.0011; 0.0018]	*0.012
	16:1	0.000062 [0.000041; 0.00014]	0.000038 [0.000031; 0.000052]	*0.018
	18:0	0.0015 [0.0012; 0.0042]	0.00097 [0.00091; 0.0014]	*0.014
	18:1	0.0015 [0.0012; 0.0032]	0.0011 [0.00095; 0.0016]	*0.017
	18:2	0.00065 [0.00034; 0.0016]	0.00032 [0.00020; 0.00043]	*0.017
	20:4	0.00029 [0.00020; 0.00066]	0.00017 [0.00015; 0.00029]	*0.036
	20:5	0.000041 [0.000022; 0.00011]	9.7e-6 [6.4e-6; 0.000018]	*0.013
	22:6	0.00022 [0.00014; 0.00048]	0.000094 [0.000080; 0.00015]	*0.0055
LPI	18:0	0.0021 [0.00069; 0.016]	0.0015 [0.00052; 0.0029]	0.085

Data are expressed as median value [25 percentile, 75 percentile], *p < 0.05.

NPSI, Neuropathic Pain Symptom Inventory; LPA, lysophosphatidic acid; LPC, lysophosphatidylcholine; LPI, lysophosphatidylinositol; LPL, Lysophospholipid; CSF, cerebrospinal fluid; LSS, lumbar spinal stenosis.

**Table 3:** Comparison of LPL levels in CSF between severe and mild group with NPSI (paresthesia/dysesthesia subscore) in the LSS patients.

		NPSI (Paresthesia/Dysesthesia subscore)		
		Severe (n = 14)	Mild (n = 14)	*p-value*
LPA	Total	0.12 [0.064; 0.29]	0.094 [0.070; 0.14]	0.095
	16:0	0.076 [0.041; 0.15]	0.054 [0.048; 0.076]	0.2
	18:0	0.0058 [0.0034; 0.0091]	0.0047 [0.0031; 0.0078]	0.42
	18:1	0.031 [0.012; 0.066]	0.021 [0.013; 0.031]	0.091
	18:2	0.014 [0.00058; 0.036]	0.0041 [0.00095; 0.0075]	0.088
	20:4	0.0060 [0.00054; 0.015]	0.0021 [0.00075; 0.0035]	*0.016
	22:6	0.00025 [0.00018; 0.0033]	0.00060 [0.00030; 0.0016]	0.33
LPC	Total	0.0086 [0.0045; 0.019]	0.0040 [0.0038; 0.0061]	*0.006
	14:0	0.00011 [0.000094; 0.00016]	0.00010 [0.000092; 0.00011]	*0.021
	16:0	0.0029 [0.0014; 0.0076]	0.0013 [0.0012; 0.0020]	*0.007
	16:1	0.000065 [0.000035; 0.00014]	0.000042 [0.000032; 0.000052]	*0.015
	18:0	0.0018 [0.00097; 0.0042]	0.00099 [0.00091; 0.0014]	*0.009
	18:1	0.0018 [0.0011; 0.0032]	0.0011 [0.00099; 0.0016]	*0.008
	18:2	0.00088 [0.00031; 0.0017]	0.00036 [0.00022; 0.00055]	*0.008
	20:4	0.00037 [0.00018; 0.00070]	0.00018 [0.00015; 0.00029]	*0.007
	20:5	0.000035 [0.000013; 0.00011]	0.000016 [7.7e-6; 0.000029]	0.055
	22:6	0.00025 [0.000089; 0.00048]	0.00011 [0.000084; 0.00016]	*0.008
LPI	18:0	0.0028 [0.00033; 0.016]	0.0011 [0.00073; 0.0024]	0.051

Data are expressed as median value [25 percentile, 75 percentile], *p < 0.05.

NPSI, Neuropathic Pain Symptom Inventory; LPA, lysophosphatidic acid; LPC, lysophosphatidylcholine; LPI, lysophosphatidylinositol; LPL, Lysophospholipid; CSF, cerebrospinal fluid; LSS, lumbar spinal stenosis.

**Table 4:** Comparison of LPL levels in CSF between severe and mild group with ZCQ in the LSS patients.

		ZCQ		
		Severe (n = 14)	Mild (n = 14)	*p-value*
LPA	Total	0.13 [0.090; 0.29]	0.075 [0.057; 0.16]	*0.037
	16:0	0.06 [0.05; 0.15]	0.051 [0.038; 0.098]	0.17
	18:0	0.0055 [0.0033; 0.0091]	0.0056 [0.0025; 0.0078]	0.41
	18:1	0.028 [0.024; 0.066]	0.013 [0.0099; 0.037]	*0.031
	18:2	0.027 [0.0029; 0.036]	0.0022 [0.00044; 0.0050]	*0.004
	20:4	0.0050 [0.0011; 0.015]	0.0011 [0.00045; 0.0030]	*0.016
	22:6	0.0016 [0.00022; 0.0049]	0.00032 [0.00018; 0.00065]	*0.016
LPC	Total	0.0064 [0.0047; 0.019]	0.0040 [0.0033; 0.0059]	*0.012
	14:0	0.00011 [0.00010; 0.00016]	0.000097 [0.000092; 0.00011]	*0.049
	16:0	0.0023 [0.0014; 0.0076]	0.0013 [0.0010; 0.0018]	*0.013
	16:1	0.000057 [0.000042; 0.00014]	0.000035 [0.000031; 0.000059]	*0.019
	18:0	0.0015 [0.0012; 0.0042]	0.00097 [0.00088; 0.0014]	*0.013
	18:1	0.0015 [0.0011; 0.0032]	0.0011 [0.00094; 0.0016]	*0.021
	18:2	0.00065 [0.00033; 0.0016]	0.00034 [0.00019; 0.00043]	*0.017
	20:4	0.0015 [0.0011; 0.0032]	0.00018 [0.00015; 0.00029]	*0.04
	20:5	0.00065 [0.00033; 0.0016]	0.000012 [6.4e-6; 0.000026]	*0.02
	22:6	0.00029 [0.00019; 0.00066]	0.000089 [0.000080; 0.00019]	*0.0097
LPI	18:0	0.0023 [0.00082; 0.016]	0.0015 [0.00041; 0.0029]	0.073

Data are expressed as median value [25 percentile, 75 percentile], *p < 0.05.

ZCQ, Zurich Claudication Questionnaire; LPA, lysophosphatidic acid; LPC, lysophosphatidylcholine; LPI, lysophosphatidylinositol; LPL, Lysophospholipid; CSF, cerebrospinal fluid; LSS, lumbar spinal stenosis.

**Table 5:** Comparison of LPL levels in CSF between severe and mild group with morphological study in the LSS patients.

		Radiography		
		Severe (n = 18)	Mild (n = 10)	*p-value*
LPA	Total	0.14 [0.090; 0.29]	0.063 [0.043; 0.092]	*0.0049
	16:0	0.091 [0.051; 0.15]	0.045 [0.031; 0.057]	*0.007
	18:0	0.0072 [0.0039; 0.0091]	0.0033 [0.0022; 0.0055]	*0.03
	18:1	0.035 [0.024; 0.059]	0.013 [0.0054; 0.021]	*0.004
	18:2	0.0065 [0.0023; 0.034]	0.0012 [0.00044; 0.0075]	0.096
	20:4	0.0043 [0.0013; 0.012]	0.00052 [0.00037; 0.0028]	*0.018
	22:6	0.00060 [0.00022; 0.0025]	0.00033 [0.00017; 0.00078]	0.31
LPC	Total	0.0064 [0.0040; 0.018]	0.0040 [0.0032; 0.0052]	*0.02
	14:0	0.00011 [0.00011; 0.00015]	0.000093 [0.000091; 0.00010]	*0.018
	16:0	0.0021 [0.0012; 0.0070]	0.0013 [0.0010; 0.0017]	*0.028
	16:1	0.000061 [0.000041; 0.00014]	0.000035 [0.000030; 0.000043]	*0.02
	18:0	0.0015 [0.00097; 0.0039]	0.00097 [0.00087; 0.0012]	*0.025
	18:1	0.0016 [0.0011; 0.0030]	0.0011 [0.00091; 0.0013]	*0.015
	18:2	0.00065 [0.00033; 0.0015]	0.00029 [0.00019; 0.00044]	*0.022
	20:4	0.00029 [0.00016; 0.00066]	0.00018 [0.00015; 0.00021]	*0.014
	20:5	0.000028 [0.000015; 0.000066]	9.7e-6 [4.5e-6; 0.000036]	0.13
	22:6	0.00018 [0.00011; 0.00036]	0.000089 [0.000078; 0.00014]	*0.028
LPI	18:0	0.0029 [0.0017; 0.011]	0.00067 [0.00031; 0.0012]	*0.03

Data are expressed as median value [25 percentile, 75 percentile], *p < 0.05.

LPA, lysophosphatidic acid; LPC, lysophosphatidylcholine; LPI, lysophosphatidylinositol; LPL, Lysophospholipid; CSF, cerebrospinal fluid; LSS, lumbar spinal stenosis.

### LPA levels in CSF of patients with LSS was correlated with corresponding LPC levels, but not with ATX levels

**Figure 1:**
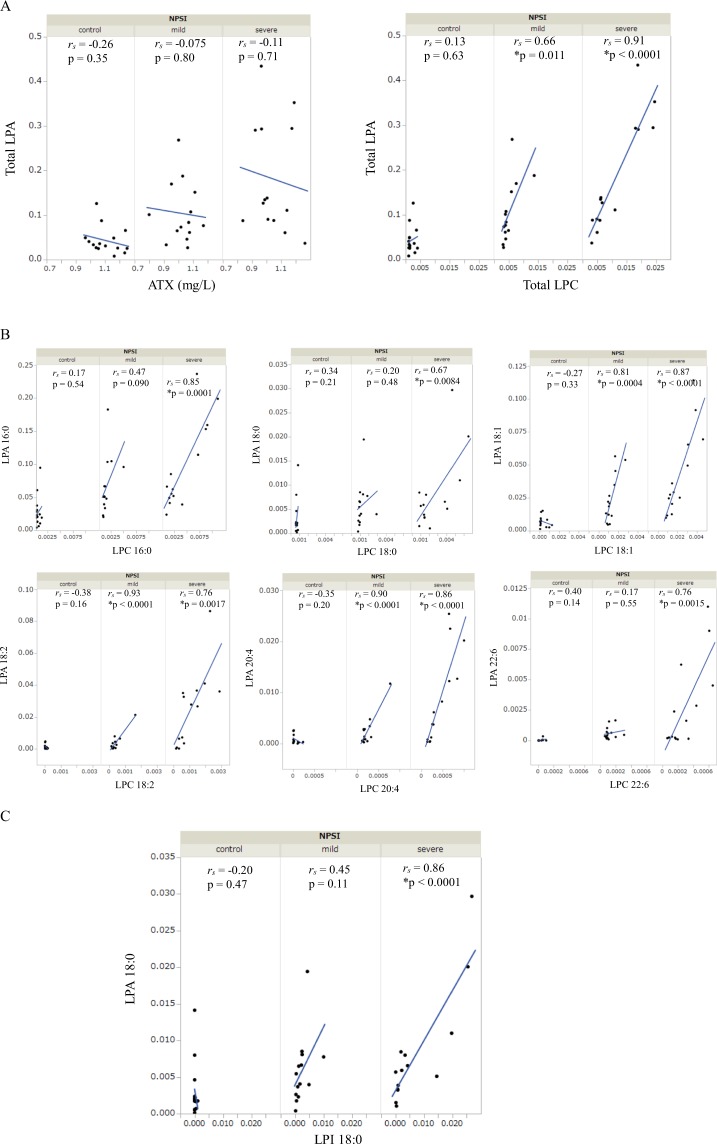
Correlation between the CSF levels of LPA and ATX or other LPLs. We respectively evaluated in controls, mild NPSI LSS group, and severe NPSI LSS group (*p < 0.05). (**A**) Correlation between the CSF levels of total LPA and ATX or total LPC. (**B**) Correlation between the CSF levels of LPA species and the corresponding LPC species. (**C**) Correlation between the CSF levels of LPA (18:0) and LPI (18:0). LPA, lysophosphatidic acid; ATX, autotaxin; LPC, lysophosphatidylcholine; NPSI, Neuropathic Pain Symptom Inventory; LSS, lumbar spinal stenosis; LPI, lysophosphatidylinositol; CSF, cerebrospinal fluid.

Since LPA is produced from LPLs other than LPA, especially LPC, via ATX, we examined the correlation between LPA and ATX or other LPLs to investigate the mechanism for the increased LPA in LSS. While there was no correlation between LPA and ATX in all groups, total LPC significantly correlated with LPA in the severe and mild NPSI LSS groups but not in the control group (Fig. 1A). In addition, three species of LPC (18:1, 18:2, and 20:4) significantly correlated with the corresponding species of LPA both in the severe and mild NPSI LSS groups but not in the control group. Three species of LPC (16:0, 18:0, and 22:6) and LPI significantly correlated with the corresponding species of LPA in the severe NPSI LSS group but not in the mild NPSI LSS and control groups (Fig. 1B,C).

### ATX antigen levels only correlated with LysoPLD activity in CSF obtained from the severe LSS group

**Figure 2:**
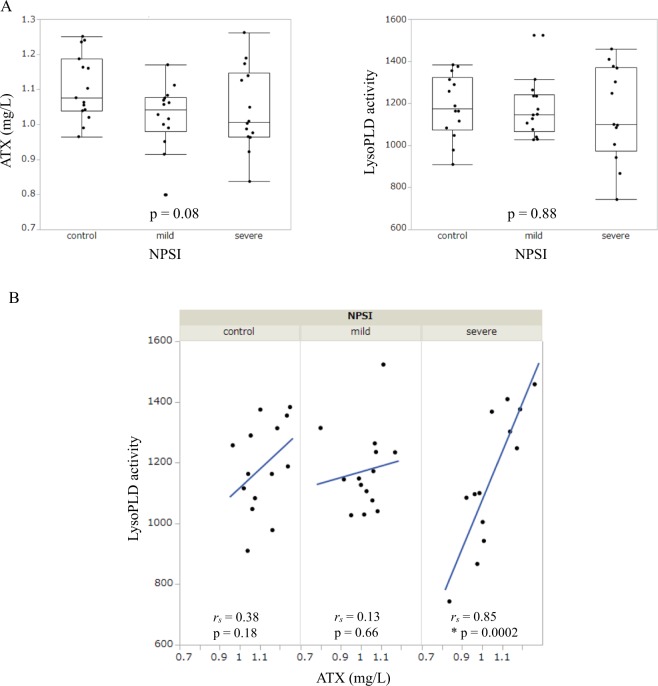
Comparison and correlation between the CSF levels of ATX and ATX activity (LysoPLD). We respectively evaluated in controls, mild NPSI LSS group, and severe NPSI LSS group (*p < 0.05). (**A**) Comparison between the CSF levels of ATX and LysoPLD. (**B**) Correlation between the CSF levels of ATX and LysoPLD. LPA, lysophosphatidic acid; ATX, autotaxin; LPC, lysophosphatidylcholine; NPSI, Neuropathic Pain Symptom Inventory; LSS, lumbar spinal stenosis; LPI, lysophosphatidylinositol; CSF, cerebrospinal fluid.

Interestingly, when we measured the CSF levels of ATX and ATX activity (LysoPLD), which strongly correlate with each other in serum^15^, the difference in the CSF levels of ATX and LysoPLD was not statistically significant among the control, mild NPSI LSS, and severe NPSI LSS groups (Fig. 2A). In contrast, a significant positive correlation between the CSF levels of ATX and LysoPLD was found in the severe NPSI LSS group (*r*_*s*_ = 0.85), whereas there was little or no correlation in the mild NPSI LSS group (*r*_*s*_ = 0.13) and the control group (*r*_*s*_ = 0.38) (Fig. 2B).

## Discussion

Our results demonstrate that most species of LPA, LPC, and LPI correlate with LSS symptom severity. Patients with LSS with severe symptoms demonstrated higher LPLs in the CSF than those with mild symptoms and controls. Different symptoms of LSS (i.e., pain, numbness, and claudication) correlated with different LPL species: only 20:4 LPA was consistently higher in patients with severe pain, numbness, and claudication. Meanwhile, almost all LPC species were higher for respective symptoms in patients with severe disease. Considering the mechanisms underlying the increased LPA in the CSF of patients with LSS, we observed that LPA levels correlated with the corresponding species of LPC but not with ATX levels, suggesting that increased LPA levels might be attributed to the increased production of LPC, possibly through enhanced phospholipase A2.

Anatomical degenerative processes of the lumbar spine are involved in the development and maintenance of clinical symptoms of LSS. The most common symptom associated with LSS is neuropathic pain radiating into the legs at rest and/or during walking, which follows anatomical compression of nerve roots and the cauda equina. Previous findings from animal neuropathic pain models demonstrate that LPA signaling is the definitive mechanism underlying neuropathic pain, in particular LPA1 and auxiliary LPA3; among LPA receptor subtypes playing a crucial role in neuropathic pain^12^, 16:0, 16:1, 18:1, 18:2, and 20:4 LPA species are the ligands predominantly responsible for LPA1 and LPA3^10,16^. This notion is partially consistent with our present findings that 20:4, 18:1, and 18:2 LPA species were closely related to LSS and with the severity of neuropathic pain and claudication. In addition to these LPAs, 22:6 LPA also showed an association with neuropathic pain and claudication severity. To our knowledge, there are no studies to support the involvement of 22:6 LPA in neuropathic pain, although 22:6 LPA levels were increased in a mouse model of spinal cord injury^17^. Anecdotally, 22:6 LPA was reported to be involved in the inflammatory process in mouse models of an allergic airway reaction and in humans with asthma^18,19^. Through a neurogenic inflammatory process, increasing 22:6 LPA might lead to the development and maintenance of neuropathic pain. Focusing on 22:6 LPA, patients with LSS with severe dysesthesia and numbness did not demonstrate increased levels of 22:6 LPA compared to those with mild symptom. Such symptoms are also common in LSS, and might arise from nerve degeneration, independent of neurogenic inflammation.

Regarding the mechanisms of LPA increase, two pathways are possible, considering the data from human plasma samples; one is that elevated ATX levels might produce increased LPA levels, as observed in cirrhosis^20^ and follicular lymphoma^21^. There is another possibility that the increased LPA might be derived from increased ATX substrates, such as LPC, as shown in acute coronary syndrome^14,22^. Considering that close correlations were only observed between LPA and the corresponding species of LPC, but not ATX, the latter mechanism is supported. As ATX levels are abundant in CSF, while LPC levels are much lower in CSF versus plasma, it is reasonable to assume that changes in LPC levels might affect LPA levels to a greater degree than ATX levels.

Different from the results/case with LPA, almost all LPC species demonstrated significantly higher concentrations in the CSF, in association with severe LSS symptoms. Although LPC can trigger demyelination and subsequently neuropathic pain^23,24^, the current understanding of LPCs in neuropathic pain is that they represent a ‘resource’ of LPA that can potentially cause and maintain both peripheral nerve injury and neuropathic pain^12,25^. Our patients with severe and mild LSS demonstrated ATX levels and its enzymatic activity comparable to controls; thus increasing LPC levels would link to increasing LPA and severity of LSS symptoms. Alternatively, LPC might impair LSS symptoms via mechanisms other than peripheral nerve injury. LPC could have an agonistic effect on the alpha-adrenergic receptor and enhance vasoconstriction^26^. LPC is also reported to induce vasoconstriction with endothelial dysfunction via nNOS uncoupling and modulating ERK1/2 activity^27^. In human study, it is reported that elevated serum LPC (20:4 and 22:6) levels is associated with ischemic stroke risk^28,29^. Degenerative LSS can lead to compression of not only nerve roots and the cauda equina but also intra-spinal vessels; this is considered to contribute to the development of neurogenic claudication in LSS: reduced arterial blood flow results in ischemia and venous congestion with compression of the nerves and secondary perfusion deficiency^30^. This ischemic and digestive mechanism would account for the typical reversibility of symptoms when patients flex the spine forward. Previous findings of a strong correlation between LPA and neuropathic pain have been elucidated using animal models with peripheral nerve injury rather than LSS. Therefore, in patients with LSS, LPC might directly impair LSS symptoms, possibly through vasoconstriction of the intra-spinal circulation.

One interesting finding of this study that remains unexplained is that ATX and its enzymatic activity were comparable between patients with LSS and controls and between patients with LSS with mild and severe symptoms; in patients with severe LSS only, ATX linearly correlated with its enzymatic activity. Such linear correlation might increase with increased severity of LSS symptoms. Considering that ATX antigen levels strongly correlate with ATX activity in plasma^15^, unknown molecules in CSF might affect ATX activity, which might be suppressed in severe LSS. Another interesting finding was that there is no evidence concerning the association between LPI and LSS, although we demonstrated increased levels in LSS. Future studies should clarify their contributions to the development and maintenance of neuropathic pain and claudication.

A limitation of this study is that the control participants were not healthy volunteers but patients with idiopathic scoliosis or bladder cancer. Since the invasive procedure was required to obtain CSF, our recruited control subjects were limited to the persons who underwent myelography or lumbar anesthesia as a part of treatment. Although the NPSI score of all control participants was 0 which means they had no painful symptoms, their clinical condition other than neuropathic pain might affect the CSF levels of LPLs. For example, it is reported that mRNA expression of LPA receptor is facilitated in the specimen with bladder cancer patients, which might elicit the slight elevation of LPL levels in CSF of control subjects^31,32^. In addition, the myelography is not always underwent particularly in institutions where the MRI imaging is valued to determine the surgery for lumbar spinal stenosis. CSF sample is difficult to obtain in such institutions. Another limitation is that the number of subjects in each group was relatively small, which might make it difficult to detect the subtle differences between groups. Our limited sample size might also affect the reliability of the spearman’s correlation analysis although we evaluated the rank correlation coefficient together with p-value. In addition, the precise demographic data of subjects are lacking in our study. The medical history of any malignancy or systemic inflammatory disease might affect the results of LPL levels in CSF. Also, there was lacking of the clinical information about the duration of the pain (acute or chronic), the use of anti-inflammatory drugs or opioids which might affect the LPA signaling. Further extensive study would be required to confirm our results.

In summary, this study demonstrated correlation among LPA and LPC and the severity of neuropathic pain caused by LSS. In addition to the proposal for LPA and LPC as treatment targets in neuropathic pain, measurement of LPA and LPC in the CSF might be an objective method to evaluate the severity of LSS.

## Methods

### Data source

We included consecutive patients with LSS who underwent myelography for the evaluation of potential spine surgery between August 2014 and March 2015. Our institution routinely performs myelography as a part of our evaluation of patients with LSS with multilevel stenosis or spondylolisthesis; this technique is used to determine the appropriate surgical procedure. Patients with concomitant spinal stenosis at other regions (i.e., cervical or thoracic spine) or previous spinal surgery were excluded. CSF samples from patients with idiopathic scoliosis or bladder cancer, who had no neurological symptoms, were used as a control group. Written informed consent was obtained from each patient. The study was approved by the institutional review board of The University of Tokyo, and was carried out in accordance with the Helsinki Declaration.

### Measurement of LPL species using LC-MS/MS

From each patient, 1 mL of CSF was collected at the time of myelography. The samples were stored at −80 °C, and the freeze-thaw treatment was limited to one time before the measurement of LPLs and ATX levels. Quantification of LPLs was performed as described by Okudaira *et al*.^33^. Briefly, the CSF samples were mixed and sonicated with a 10-fold volume of methanol and an internal standard. After centrifugation at 21,500 × g, the resulting supernatant was recovered and used for LC-MS analysis. Then, 20 µL of methanol extract was separated using Nanospace LC (Shiseido) equipped with a C18 CAPCELL PAK ACR column (1.5 × 250 mm; Shiseido) using a gradient of solvent A (5 mM ammonium formate in water) and solvent B (5 mM ammonium formate in 95% [v/v] acetonitrile). Elution was sequentially ionized using an ESI probe, and the parent ion (m/z 380.2) and fragment ion (m/z 264.2) were monitored in the positive mode using a Quantum Ultra Triple Quadrupole Mass Spectrometer (Thermo Fisher Scientific). For each lysophospholipid class, 12 acyl chains (14:0, 16:0, 16:1, 18:0, 18:1, 18:2, 18:3, 20:3, 20:4, 20:5, 22:5, and 22:6) were monitored. In the present study, we extracted lipids under a neutral condition, and we did not separate 1-acyl-2-lyso- and 2-acyl-1-lyso-phospholipids. We calculated the concentrations of lysophospholipids from the area ratio to the internal standard: 1 µM 17:0 LPA (for LPA, lysophosphatidylethanolamine, LPI, lysophosphatidylglycerol, and lysophosphatidylserine species) or 10 µM 17:0 LPC (for LPC species).

### Measurement of ATX levels in the CSF

The ATX antigen levels in the CSF were determined using a two-site immunoenzymetric assay with an ATX assay reagent and the TOSOH AIA system (TOSOH, Tokyo, Japan)^15^.

### LysoPLD activity assay

The lysoPLD activity in CSF samples was assessed based on the amount of choline released using LPC as the substrate, as described previously^22^.

### Clinical and magnetic resonance imaging evaluation

The Neuropathic Pain Symptom Inventory (NPSI)^34^ and the Zurich Claudication Questionnaire (ZCQ)^35^ were used to assess the clinical severity of LSS. We differently calculated the NPSI subscore of paresthesia/dysesthesia by adding Q11 and Q12 in the NPSI. On the basis of the median value, the patients were categorized into two groups according to symptom severity and pain, grading for respective clinical symptoms. Axial magnetic resonance imaging was used to evaluate the morphological severity of LSS, and patients were classified into two groups (grade A/B or grade C/D) based on morphological grading (using the CSF/rootlet ratio)^1^. Briefly, rootlets can be individualized in Grade A/B whereas no rootlets can be recognized in Grade C/D with axial T2 magnetic resonance imaging.

### Statistical analyses

All statistical analyses were performed using JMP 12 (SAS Institute, Cary, NC, USA). The CSF levels of undetectable LPLs were treated as 0 for statistical analysis. To examine the association between CSF levels of LPLs and the severity of LSS, an unpaired two-tailed Student’s t-test following the F-test was used with the comparison of two groups, and the analysis of variance was used with the comparison among three groups. Correlations between LPA and ATX or other LPLs were investigated using the Spearman correlation test. We also used the Spearman correlation test to evaluate the correlation between ATX and LysoPLD. In all statistical analyses, values were considered significant at P < 0.05.

## Supplementary information

Supplementary Figure S1
