# Supplementary material for: Lysophosphatidic acids and their substrate lysophospholipids in cerebrospinal fluid as objective biomarkers for evaluating the severity of lumbar spinal stenosis
Source: Sci Rep. 2019 Jun 24;9:9144. doi: 10.1038/s41598-019-45742-7 (PMC6591408; doi:10.1038/s41598-019-45742-7)
Supplement: Supplementary file 1 — Supplementary Figure S1 [file 41598_2019_45742_MOESM1_ESM.pdf]

Lysophosphatidic acids and their substrate lysophospholipids in cerebrospinal fluid as objective biomarkers for evaluating the severity of lumbar spinal stenosis

Kentaro Hayakawa[1], Makoto Kurano[2], Junichi Ohya[1], Takeshi Oichi[1], Kuniyuki Kano[3], Masako Nishikawa[2], Baasanjav Uranbileg[2], Ken Kuwajima[4], Masahiko Sumitani[4,5], Sakae Tanaka[1], Junken Aoki[3], Yutaka Yatomi[2], Hirotaka Chikuda[6]

[1]Department of Orthopaedic Surgery, The University of Tokyo, Faculty of Medicine, Tokyo, Japan
[2]Department of Clinical Laboratory Medicine, The University of Tokyo Hospital, Tokyo, Japan
[3]Graduate School of Pharmaceutical Sciences, Tohoku University, Molecular and Cellular Biochemistry, Sendai, Japan
[4]Department of Anesthesiology and Pain Relief Center, The University of Tokyo Hospital, Tokyo, Japan
[5]Department of Pain and Palliative Medicine, The University of Tokyo Hospital, Tokyo, Japan
[6]Department of Orthopaedic Surgery, Gunma University, Graduate School of Medicine, Maebashi, Japan

Supplementary Figure S1

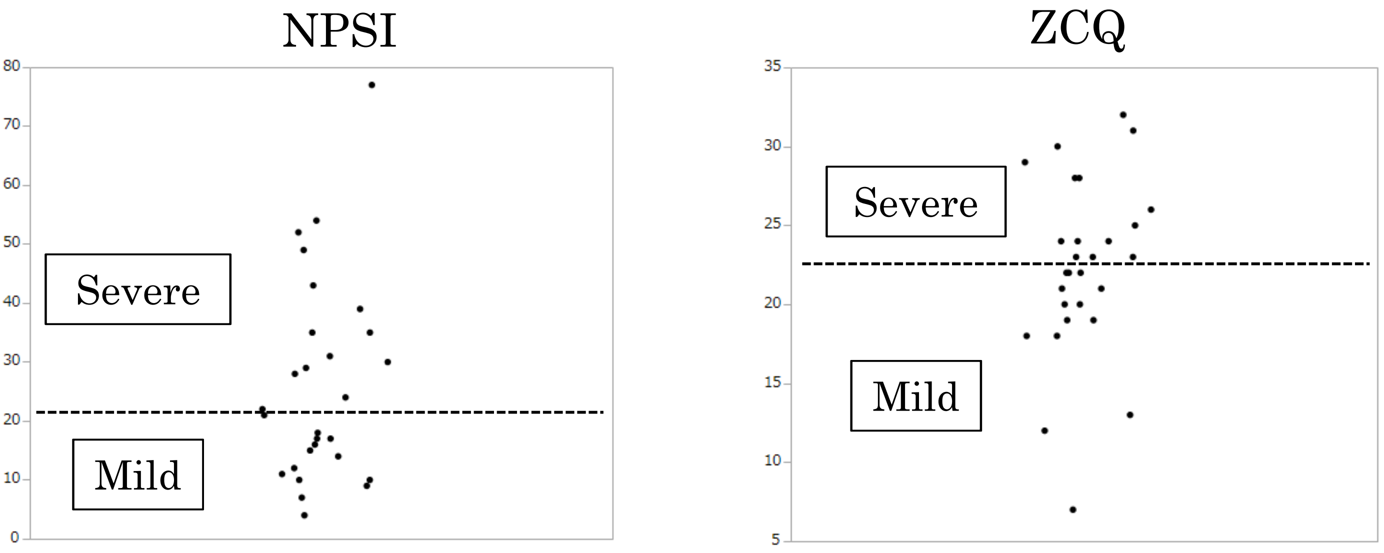

Supplementary Figure S1: Distribution of NPSI and ZCQ scores. Patients were categorized into two groups as Mild/Severe.

NPSI, Neuropathic Pain Symptom Inventory; ZCQ, Zurich Claudication Questionnaire
